# A novel imatinib analogue inhibitor of chronic myeloid leukaemia: design, synthesis and characterization—explanation of its folded conformation

**DOI:** 10.1098/rsos.241654

**Published:** 2025-01-29

**Authors:** Rodolfo Moreno-Fuquen, Juan F. Avellaneda-Tamayo, Kevin Arango-Daraviña, Javier Ellena, Alan R. Kennedy

**Affiliations:** ^1^ Grupo de Cristalografía, Departamento de Química, Universidad del Valle, Calle 13 Carrera 100, Santiago de Cali, 760042, Colombia; ^2^ DIFACQUIM Research Group, Department of Pharmacy, School of Chemistry, Universidad Nacional Autónoma de México, Avenida Universidad 3000, Mexico City, 04510, Mexico; ^3^ Instituto de Física de São Carlos, Universidade de São Paulo, USP, Avenida Trabalhador São-carlense 400, Parque Arnold Schmidt, CEP 13566-590, São Carlos, SP, Brazil; ^4^ WestCHEM, Department of Pure and Applied Chemistry, University of Strathclyde, 295 Cathedral Street, Glasgow G1 1XL, UK

**Keywords:** density functional theory, molecular electrostatic potential, Hirshfeld surface, energy framework, molecular docking, chronic myeloid leukaemia

## Abstract

Chronic myeloid leukaemia (CML) is primarily treated using imatinib mesylate, a tyrosine kinase inhibitor (TKI) targeting the BCR::ABL1 oncoprotein. However, the development of drug resistance and adverse side effects necessitate the exploration of alternative therapeutic agents. This study presents the synthesis and characterization of a novel imatinib analogue, 3-chloro-*N*-(2-methyl-5-((4-(pyridin-2-yl)pyrimidin-2-yl)amino)phenyl)benzamide (PAPP1). The compound’s structure was elucidated using X-ray crystallography and spectroscopic techniques, including NMR, infrared and UV–visible. Crystallographic analysis reveals that PAPP1 consists of a phenyl–amino–pyridine–pyrimidine (PAPP) scaffold with substituted aromatic rings forming a nearly coplanar geometry. Additionally, supramolecular interactions in the crystal are mediated by hydrogen bonds and dispersion forces, forming dimers and layered structures. Molecular docking studies demonstrate strong binding affinity to the ABL1 enzyme, with PAPP1 showing comparable binding energy to imatinib, indicating its potential as a lead compound for further development. Computational studies, including molecular electrostatic potential and vibrational analysis, provide further support for the structural stability and bioactivity of PAPP1. These findings suggest that PAPP could be a promising scaffold for future CML drug design, offering a potential alternative to existing TKIs, and PAPP1 is a promising lead susceptible to optimization.

## 1. Introduction

The pressing need to discover new drugs for treating various diseases drives the rational development of molecularly targeted compounds. This is especially true in anticancer treatments, where tyrosine kinase (TK) enzymes play a crucial role. TKs are a large family of membrane-bound and intracellular enzymes responsible for initiating signalling cascades by phosphorylating proteins within the cell [1]. In the context of specific diseases, these enzymes often become dysregulated, leading to metabolic or reproductive disruptions and, in some cases, apoptosis [2].

Over the past few decades, the inhibition of TKs has emerged as an essential therapeutic strategy, particularly for chronic myeloid leukaemia (CML) [3]. CML is characterized by a chromosomal translocation between chromosomes 9 and 22, resulting in the formation of the BCR::ABL1 oncoprotein, which drives the uncontrolled proliferation of blood stem cells [4,5]. Imatinib mesylate was the pioneering drug targeting this oncoprotein, marking a breakthrough in CML treatment due to its ability to inhibit the BCR::ABL1 TK domain [6,7]. However, long-term use of imatinib has revealed several side effects, including cardiotoxicity [8], dermatological issues, and hepatic and pancreatic dysfunctions [9], in addition to the development of resistance in 15−20% of patients [10].

To address these challenges, second- and third-generation TK inhibitors (TKIs), such as nilotinib and ponatinib, were developed [11]. However, adverse effects persist. As a result, the search for novel compounds that target TKs with improved efficacy and fewer side effects continues [12,13].

The phenyl–amino–pyridine–pyrimidine (PAPP) scaffold has emerged as a promising molecular framework in pharmacological research, effectively treating various malignancies, including CML and breast cancer [14]. Several research groups have explored the PAPP scaffold as the core structure for designing new TKIs. These studies aim to identify structural modifications that improve the therapeutic profile of TKIs [15,16].

**Figure 1.**
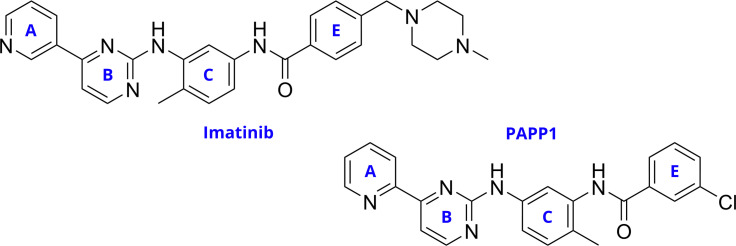
Structural modifications proposed from imatinib to PAPP1 (D is omitted because it is not affected by those modifications).

**Figure 2.**
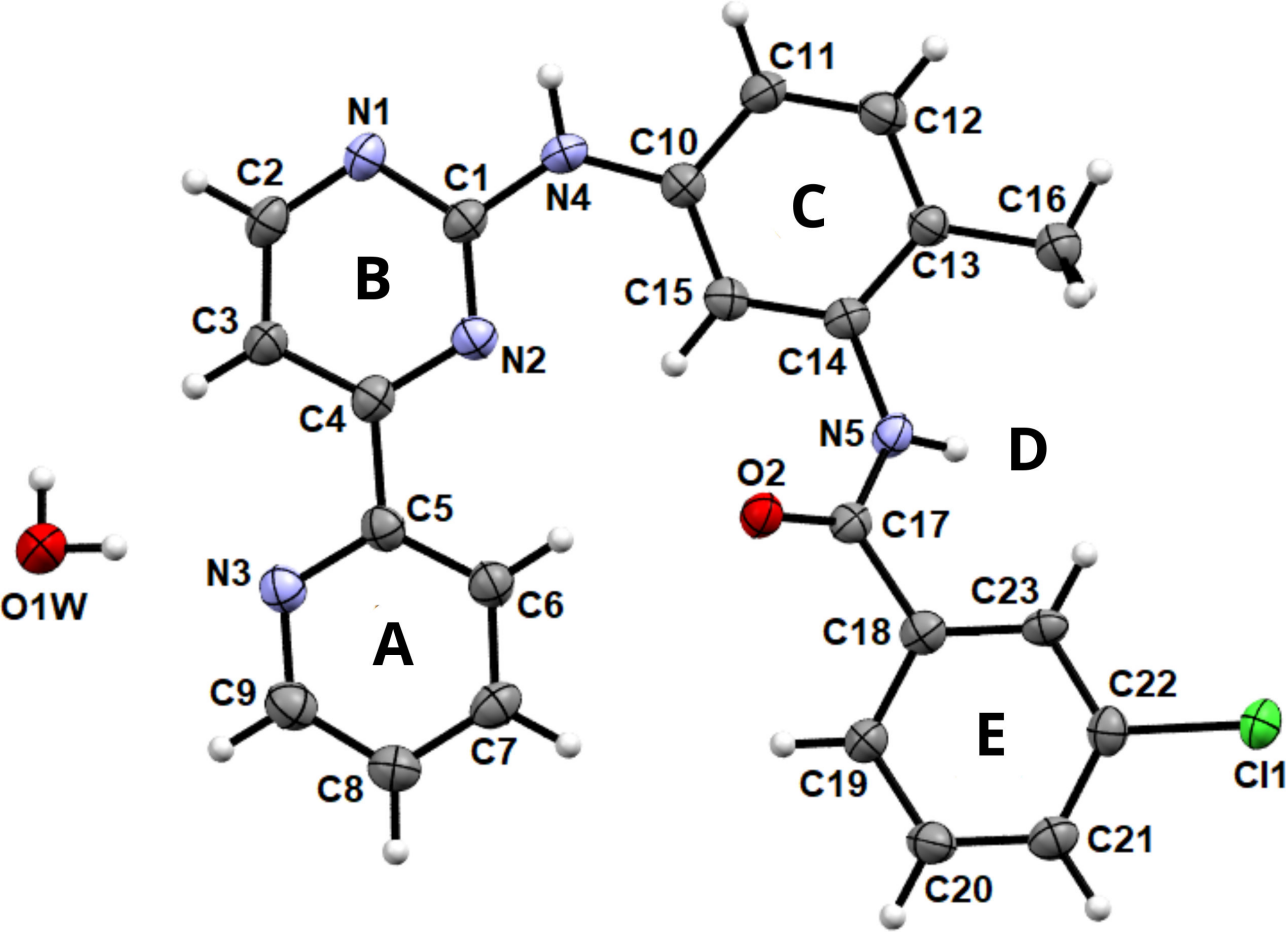
Molecular structure of PAPP1 with anisotropic thermal vibration ellipsoids drawn at the 50% probability level. The hydrogen atoms are shown as spheres of arbitrary radius.

This study focuses on synthesizing and characterizing a new PAPP-type compound, 3-chloro-*N*-(2-methyl-5-((4-(pyridin-2-yl)pyrimidin-2-yl)amino)phenyl)benzamide (PAPP1). This compound was designed with specific structural variations, including the repositioning of the heteroatom in the pyridine ring A, a methyl group shift on phenyl ring C, and the replacement of the piperazinyl ring with an electron-attracting group on benzyl ring E (figures 1 and 2). These modifications are intended to enhance the efficacy of the compound and interaction with TK enzymes, specifically targeting CML-related proteins, emphasizing the potential presence of halogen bonds relevant to ligand–protein interactions.

## 2. Results and discussion

The design and synthesis of TKIs have been central to addressing challenges in CML treatment. In this study, we developed PAPP1, a compound structurally analogous to imatinib and nilotinib, with essential modifications in the pyrimidine and pyridine rings to improve bioavailability and protein–ligand interactions.

These structural changes involved the shift of the nitrogen atom in the pyridine ring A from the *meta* to the *ortho* position, which was hypothesized to increase the hydrogen bonding capacity, and the replacement of the piperazine ring with a more electron-withdrawing group to improve the protein–ligand interactions and pharmacokinetics (ring E in figure 1) [17].

### 2.1. Molecular and crystal structure

Hemihydrated PAPP1 crystallized in the orthorhombic space group *Pbcn*, and its molecular structure was elucidated by X-ray diffraction. The compound consists of four key rings (figure 2): pyridine (A), pyrimidine (B), and two aromatic C6 rings (C and E), substituted with a methyl group and a chlorine atom, respectively. The structural geometry revealed slight torsion between the pyrimidine ring (B) and the other rings (from A by 16.28(18)°, from C by 8.92(19)° and from E by 9.06(19)°), suggesting nearly coplanar behaviour, which could enhance the compound’s binding potential in biological systems [18]. The amido group (D) forms a distinct angle with the (B) plane by 41.30(15)°, further influencing molecular conformation. These structural insights are crucial for understanding the interaction dynamics between PAPP1 and target proteins.

Data collection and crystal refinement details are summarized in electronic supplementary material, table S1. The bond lengths and angles in the crystal structure were consistent with typical bond metrics (electronic supplementary material, table S2).

### 2.2. Hydrogen bonding and supramolecular features

Supramolecular interactions, specifically hydrogen bonding, significantly stabilize the PAPP1 crystal structure. In the supramolecular analysis of PAPP1, an intramolecular C_6_–H_6_∙∙∙O_2_ bond plays a crucial role in maintaining energetic stability and ensuring the molecule’s folded conformational structure. Intermolecular interactions, such as the N5–H5N and C16–H16(2) hydrogen bond donor (HBD) groups interacting with the O_2_^a^ atom at (*x*, 2 − *y*, ½ + *z*), stabilize relatively strongly and act as pillars between molecular layers (table 1).

**Table 1.** Hydrogen bond geometry (Å, °) and other weaker interactions.

D–H∙∙∙A	D–H	H∙∙∙A	D∙∙∙A	D–H∙∙∙A
N5–H5N∙∙∙O2^a^	0.86(4)	2.10(4)	2.958(4)	169(4)
C16–H16(2)∙∙∙O2^a^	0.98	2.52	3.352(5)	143.2
O1w–H1w∙∙∙N3^b^	0.89(4)	2.11(4)	2.981(4)	166(4)
N4–H4N∙∙∙N1^c^	0.89(4)	2.40(4)	3.273(4)	166(4)
C6–H6∙∙∙O2	0.95	2.60	3.455(5)	150
C22–Cl1^b^∙∙∙Cg1			3.5057(19)	

^a^ *x*, 2 − *y*, ½ + *z*.

^b^ 2 − *x*, *y*, 3/2 − *z*.

^c^ 2 − *x*, 2 − *y*, 1 − *z*.

**Figure 3.**
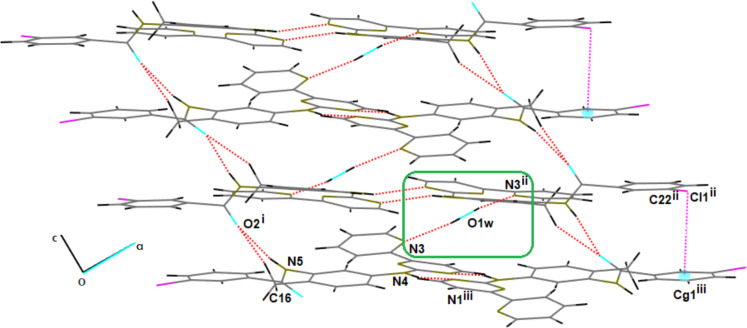
The crystal structure of PAPP1 shows hydrogen and halogen bonding interactions, forming dimer chains along [301].

Additionally, the N4–H4N group forms hydrogen bonds with the N1^c^ atom at (2*x*, 2 − *y*, 1 − *z*), facilitating dimerization and promoting crystal growth stability. The O1w–H1w group acts as a HBD for N3^b^ at (2*x*, *y*, 3/2 − *z*), bridging different molecular layers (figure 3) and leading to the formation of fused R^4^_4_(28) and R^5^_5_(32) ring motifs that propagate along the [001] direction. The supramolecular stability of PAPP1 is further reinforced by halogen bonding between C22–Cl1^b^ and Cg1^c^, which supports layer stability and facilitates the formation of R^2^_2_(10) rings.

The crystal growth behaviour of PAPP1 resembles that observed in the α-conformation of imatinib mesylate, where dimer chains are formed through hydrogen-bonding interactions with the mesylate ion [19].

### 2.3. Hirshfeld surface analysis

The Hirshfeld surface (HS) analysis of PAPP1 was conducted to investigate the intermolecular interactions that contribute to crystal formation. A two-dimensional fingerprint plot analysis was performed to quantify the partial contributions of these interactions. This analysis maps the *d*_norm_ values by studying the contact distances *d*_e_ (external atoms) and *d*_i_ (internal atoms) from the HS to the nearest atoms inside and outside the molecular boundary. When the *d*_norm_ values are less than the sum of the van der Waals (vdW) radii, the surface is highlighted in red; higher values result in a blue surface, while values close to the vdWr sum appear white [20]. This mapping reveals the critical role of hydrogen bonding and weaker interactions, such as vdW forces, in stabilizing the crystal growth process (table 1). These interactions were analysed using CrystalExplorer software [21].

**Figure 4.**
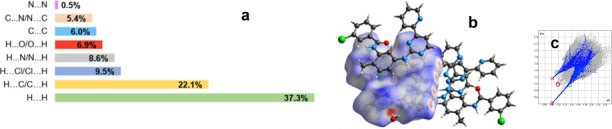
(*a*) HS calculated for PAPP1 on *d*_norm_. (*b*) Its fingerprint. The circles in grey mark the H∙∙∙N/N∙∙∙H, contacts, in red H∙∙∙O/O∙∙∙H, in golden H∙∙∙C/C∙∙∙H and in green H∙∙∙H and (*c*) relative contribution to the HS for key intermolecular contacts in crystal growth of PAPP1.

The HS analysis revealed that the O1w–H1w∙∙∙N1^b^ hydrogen bonding interactions, identified by large, bright red spots on the *d*_norm_ surface, are the primary intermolecular forces stabilizing the molecular layers (figures 3 and 4*a*). Additionally, N4–H4N∙∙∙N1^c^ interactions, which form dimers across the HS, appear as two less intense red spots and contribute 7.0% to the total HS for H∙∙∙N/N∙∙∙H interactions. Parallel to these red regions, two other areas are observed, representing C∙∙∙H/H∙∙∙C interactions, which further strengthen dimer formation, contributing 21.4% to the total HS. A side view of the HS reveals red spots originating from O2, branching into two interactions, N5–H5∙∙∙O2^a^ and C16–H16(2)∙∙∙O2^a^, which display O∙∙∙H/H∙∙∙O contacts, contributing 11.7% to the total HS (figure 4*b*,*c*).

Although C6–H6∙∙∙O2 interactions are crucial for the folded conformational structure of PAPP1, O∙∙∙H/H∙∙∙O interactions are not visible on the HS due to their intramolecular nature. The two-dimensional fingerprint for O∙∙∙H/H∙∙∙O interactions shows non-symmetric behaviour (figure 4*b*) because the two oxygen atoms in the structure have different roles, with O2 participating in both intra- and intermolecular interactions. This imbalance leads to a higher contribution of O2 to the HS, as evidenced by a more pronounced fingerprint. The non-symmetric fingerprint in the clamp region shows H∙∙∙O/O∙∙∙H interactions at 7.7%, with *d*_e_ = 0.4 Å and *d*_i_ = 0.8 Å. In comparison, O∙∙∙H/H∙∙∙O interactions account for 3.9%, with *d*_e_ = 1.18 Å and *d*_i_ = 0.8 Å.

Weak π∙∙∙π interactions, expressed as C∙∙∙C contacts, represent 6.3% of the total HS, indicating stacking interactions between molecular layers. C∙∙∙N/N∙∙∙C interactions contribute 5.2%, with *d*_i_ and *d*_e_ distances exceeding 1.6 Å. H∙∙∙Cl/Cl∙∙∙H interactions contribute 9.1% to the HS, with *d*_e_ = 1.1 Å and *d*_i_ = 1.7 Å. Additional π∙∙∙π interactions, manifested as halogen-Cl∙∙∙π bonds, represent 2.9% of the total HS. The most significant contributors to the HS are H∙∙∙C/C∙∙∙H and non-bonding H∙∙∙H interactions, which account for 21.4 and 36.1%, respectively (figure 4*c*).

### 2.4. Analysis of the molecular electrostatic potential

**Figure 5.**
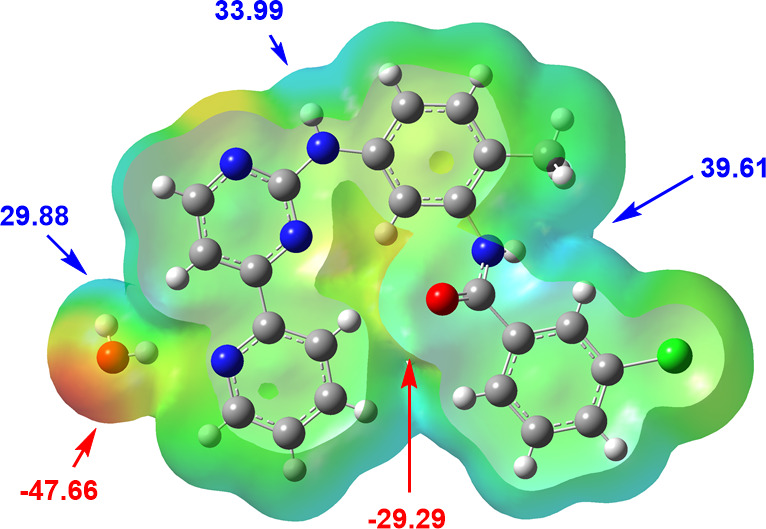
The MEP surface of PAPP1 in kcal mol^−1^.

To elucidate the intermolecular contacts observed in PAPP1, we analysed its molecular electrostatic potential (MEP) surface (figure 5). The MEP represents the electric field experienced by a positive charge across the molecular surface, highlighting regions susceptible to nucleophilic attacks (maxima in blue) or electrophilic additions (minima in red). This analysis aids in characterizing the potential electrostatic interactions between PAPP1 and surrounding entities, either within the crystal structure or in biological systems, particularly in active sites of biomolecules. Hydrophobic interactions were identified in regions populated by slightly polarized hydrogen atoms, such as those in aromatic rings, suggesting potential interactions with biomolecules [22]. The quantitative assessment of MEP was performed using Multiwfn 3.6 [23].

The regions that show higher susceptibility to nucleophilic attack or hydrogen bonding are located on electronegative atoms such as nitrogen and oxygen. The global minimum in the potential energy is located near the oxygen atom of a water molecule, with a value of −47.66 kcal mol^−1^. Additional significant minima were identified around the oxygen atom of the amide group, with values of −29.29 and −16.82 kcal mol^−1^, situated both in front of and behind the molecular surface (figure 5). These minima help stabilize the repulsion between oxygen atoms on opposing surfaces, restricting molecular rotation and preventing an extended conformation. The most electropositive regions are concentrated around the protons of the amido and amino groups, with potentials of 39.61 and 33.99 kcal mol^−1^, respectively. Additionally, the Hw protons generate positive potentials of 29.88 kcal mol^−1^ near the O1w oxygen atom (figure 5). This configuration facilitates the rotation of the C4–C5 bond, impacting the rotation of the pyridine ring and forming an intramolecular O1w–H1w∙∙∙N3 hydrogen bond. This interaction effectively reduces the negative charge in the central region of the molecule, stabilizing the folded conformation of PAPP1.

In comparison, an analysis of the MEP of imatinib mesylate revealed similar electropositive regions near the amino and amido groups, with values of 39.10 and 30.61 kcal mol^−1^, respectively. A global minimum of −47.98 kcal mol^−1^ was observed near the oxygen atom of the amide group and the nitrogen atom of the pyridine ring, with another significant minimum at −42.72 kcal mol^−1^. In PAPP1, the potential around the pyridine nitrogen participates in forming an O1w–H1w∙∙∙N3^b^ hydrogen bond, mediated by the proximity of a water molecule. This interaction potentially hinders a further engagement of the pyridine nitrogen with protein residues in a hypothetical protein–ligand complex.

The positioning of the methyl group in imatinib plays a critical role in its molecular conformation. Factors such as the group’s induction effect, its spatial volume, and the absence of strong intramolecular forces between the (A) and (E) rings (figures 1 and 2) contribute to the molecule’s extended conformation. In contrast, in the imatinibium dipicrate system [24], the formation of an N–H∙∙∙N-type bond induces the formation of R^2^_2_(24) rings, along with the stacking of (E) rings from different imatinib molecules, ultimately promoting its folded conformation. Strong hydrogen bonding between the picric acid and piperazine moieties further anchors and stabilizes this conformation.

In PAPP1, however, the methyl group is repositioned from an *ortho* to a *para* location, diminishing its influence on the molecular conformation. A water molecule within the PAPP1 crystal structure introduces a distinct structural behaviour, facilitating the formation of robust hydrogen bonds around the water molecule, which act as an anchor. This anchoring effect promotes a quasi-planar arrangement of the (A), (B), and (C) rings. The water-mediated interactions between molecules across layers are critical in stabilizing the folded structure of PAPP1.

### 2.5. Energy frameworks

To further understand the crystallization process of PAPP1, we conducted calculations of intermolecular interactions interpreted in terms of molecular pair interaction energies [25]. These calculations were based on X-ray diffraction data, with energy components expressed as *E*_ele_ (classical electrostatic energy), *E*_rep_ (repulsion energy), *E*_disp_ (dispersion energy), and *E*_pol_ (polarization energy). The supramolecular energy networks responsible for the crystal architecture were analysed using CrystalExplorer software, which applies the PIXEL model for interpreting non-covalent interactions [26]. The interaction energies for PAPP1 were derived from density functional theory (DFT) calculations using the B3LYP-D3/6-31G method, which incorporates Grimme’s D3 correction to account for dispersion effects [27]. Only interactions with neighbouring molecules within the first coordination sphere at distances of less than 3.8 Å were considered (table 2).

**Table 2.** Non-covalent interaction energy components (kJ mol^−1^) for PAPP1.

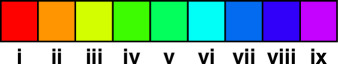							
Colour	*N*	*R*	*E*_Ele_	*E*_Pol_	*E*_Dis_	*E*_Rep_	*E*_Tot_
i	1	7.89	−33.4	−7.5	−9.7	35.6	−27.3
ii	2	4.90	−48.9	−12.4	−120.3	100.2	−103.6
iii	2	10.63	−4.5	−1.5	−23.7	20.8	−13.7
iv	2	13.30	−1.9	−0.1	−5.9	5.4	−3.9
v	1	12.19	−45.7	−8.9	−29.4	60.2	−43.2
vi	1	10.39	−0.3	−1.0	−38.1	16.6	−23.9
vii	2	11.28	−3.1	−0.7	−21.3	14.5	−13.5
viii	2	9.86	−5.9	−2.3	−22.3	13.9	−18.7
ix	1	8.48	−5.2	−1.9	−4.7	4.8	−8.0

The scaling factors of the electron densities to determine *E*_Tot_: *E*_Ele_ = 1.057, *E*_Pol_ = 0.740, *E*_Dis_ = 0.871 and *E*_Rep_ = 0.618. *N* refers to the number of molecules sharing the symmetry and equivalent interaction energies, and *R* is the distance between molecular centroids (Å).

**Figure 6.**
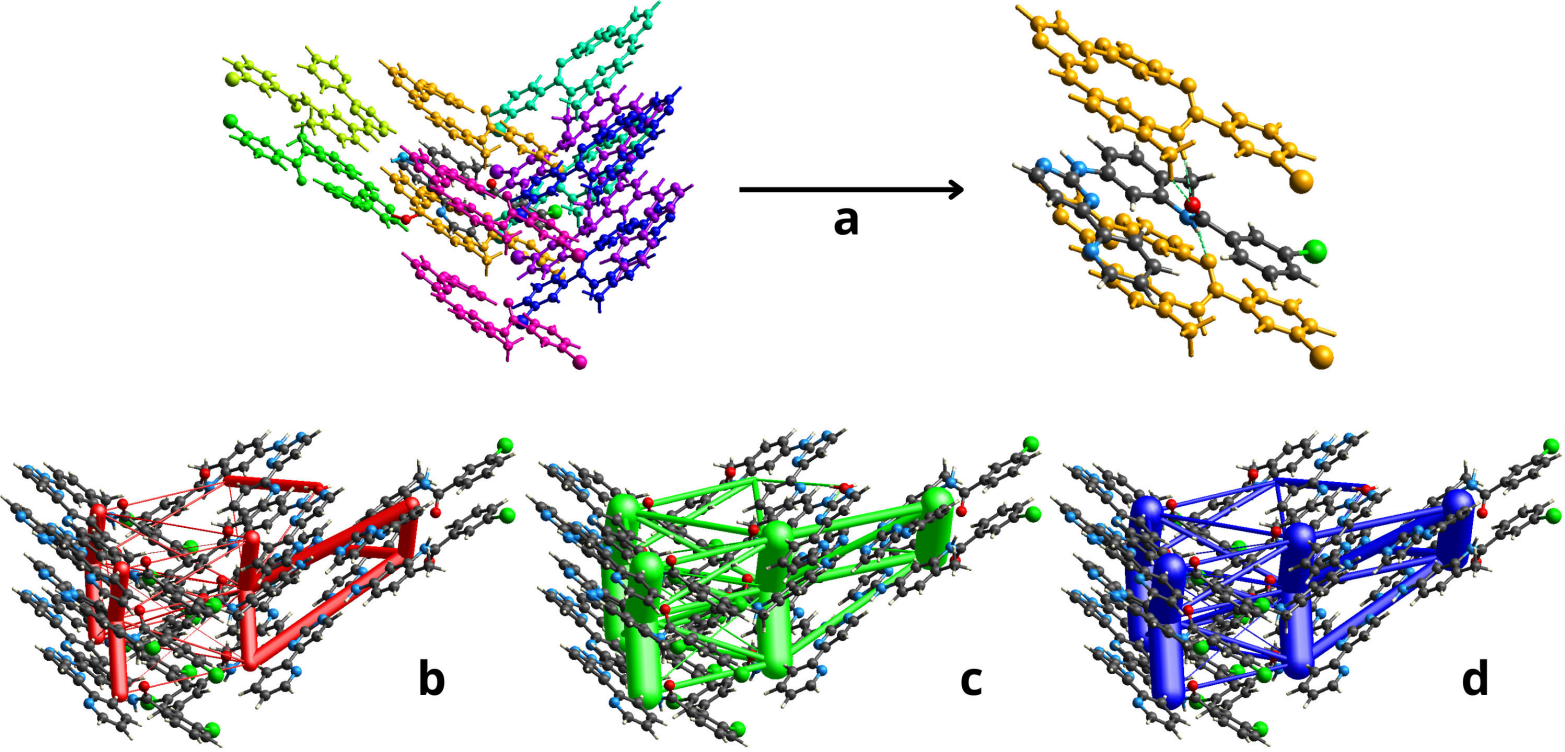
(*a*) Arrangement of molecules in the first coordination sphere. Diagrams of interaction energy networks in their (*b*) electrostatic, (*c*) dispersion and (*d*) total components.

The strongest interaction, with a total energy of −103.6 kJ mol^−1^, occurs between the molecule in the asymmetric unit and those in the adjacent dimeric layers (orange colour, ii, figure 6*a*), with centroids separated by 4.90 Å. These interactions are characterized by overlapping aromatic rings, which contribute significantly to the overall energy: −120.3 kJ mol^−1^ from dispersion, −48.9 kJ mol^−1^ from electrostatic attraction and 100.2 kJ mol^−1^ from repulsion. The substantial dispersion contribution is attributed to π∙∙∙π stacking interactions between the aromatic rings, while the electrostatic attraction results from hydrogen bonding between the layers, specifically N(5)–H(5N)∙∙∙O(2)^i^ and C(16)–H(16)∙∙∙O(2)^i^. The elevated repulsion energy is explained by the proximity of the dimers, with interatomic distances below the vdWr.

The second most significant interaction has a total energy of *E*_Tot_ = −43.2 kJ mol^−1^, occurring between the molecules that form the dimer (light green, v). This interaction is predominantly electrostatic, with a contribution of −45.7 kJ mol^−1^, mainly associated with N(4)–H(4N)∙∙∙N(1)^iii^ hydrogen bonds.

The interaction with the water molecule in the asymmetric unit has a total energy of *E*_Tot_ = −27.3 kJ mol^−1^. The main contributions to this interaction are *E*_ele_ = −33.4 kJ mol^−1^, arising from the O(1 w)–H(1w)∙∙∙N(3)^i^ hydrogen bond, and *E*_rep_ = 35.6 kJ mol^−1^, due to the proximity of the water molecule to the aromatic hydrogen atoms of the heterocyclic rings.

The interaction energy networks between molecular pairs are illustrated in figure 6, showing the direction and magnitude of the interactions across their electrostatic (figure 6*b*), dispersion (figure 6*c*), and total energy (figure 6*d*) components. These interactions are visually represented by cylinders whose thickness corresponds to the magnitude of the intermolecular energies.

Overall, it was observed that dispersion interactions (*E*_DisTotal_ = −277.5 kJ mol^−1^) contribute most significantly to the intermolecular framework, followed by electrostatic interactions (*E*_EleTotal_ = −144.8 kJ mol^−1^), thereby playing a central role in stabilizing the crystal structure (table 2).

### 2.6. HOMO–LUMO analysis

**Figure 7.**
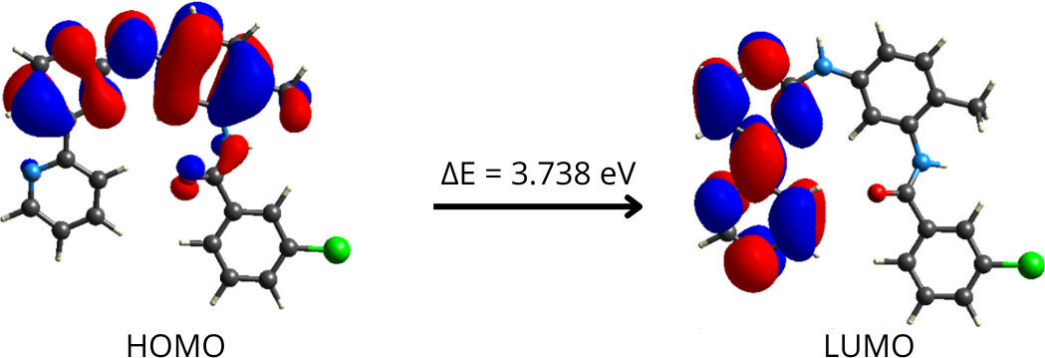
HOMO–LUMO transition energies and wave function of PAPP1 using the DFT method with the B3LYP functional and 6-31G(d,p) basis set.

A computational study using DFT/B3LYP functional and 6-31G(d,p) basis set, implemented in the CrystalExplorer program and using the TONTO system for quantum crystallography, was performed to investigate the molecular geometry and electronic properties of PAPP1. This analysis focused on the frontier molecular orbitals, specifically the highest occupied molecular orbital (HOMO) and the lowest unoccupied molecular orbital (LUMO), to gain deeper insight into the electronic characteristics of the molecule. Theoretical UV–visible spectroscopy and frontier orbital analysis revealed a minimum π–π* electronic transition energy of 3.738 eV for PAPP1, compared with 3.776 eV for imatinib in its commercial formulation (figure 7) [28]. Molecules with lower electronic transition energies typically exhibit higher chemical reactivity, as their frontier electrons and molecular orbitals are better arranged for interaction with biomolecules [29].

The PAPP1 system, characterized by a relatively small HOMO–LUMO energy gap and a nearly planar arrangement of the A, B, and C rings, facilitates electronic transitions to nearby energy levels, thereby increasing its chemical reactivity. In this system, the HOMO is distributed across the (B) and (C) rings, as well as the amino group and the nitrogen and oxygen atoms of the amido group. Conversely, the LUMO is predominantly located over the (A) and (B) rings.

### 2.7. Protein–ligand interaction through molecular docking

Molecular docking is an advanced computational technique used to predict the structure of ligand–receptor complexes by estimating the strength of non-covalent interactions within the active site [30]. This approach assessed the potential interaction between PAPP1 and the TK domain of the ABL1 protein. Inhibition of this active site is crucial for mitigating the effects of the BCR::ABL1 mutation in CML. Information regarding the interactions within the active site was obtained from the Protein Data Bank (PDB code: 1IEP), which contains the crystal structure of the ABL1 kinase domain in complex with imatinib [31].

**Figure 8.**
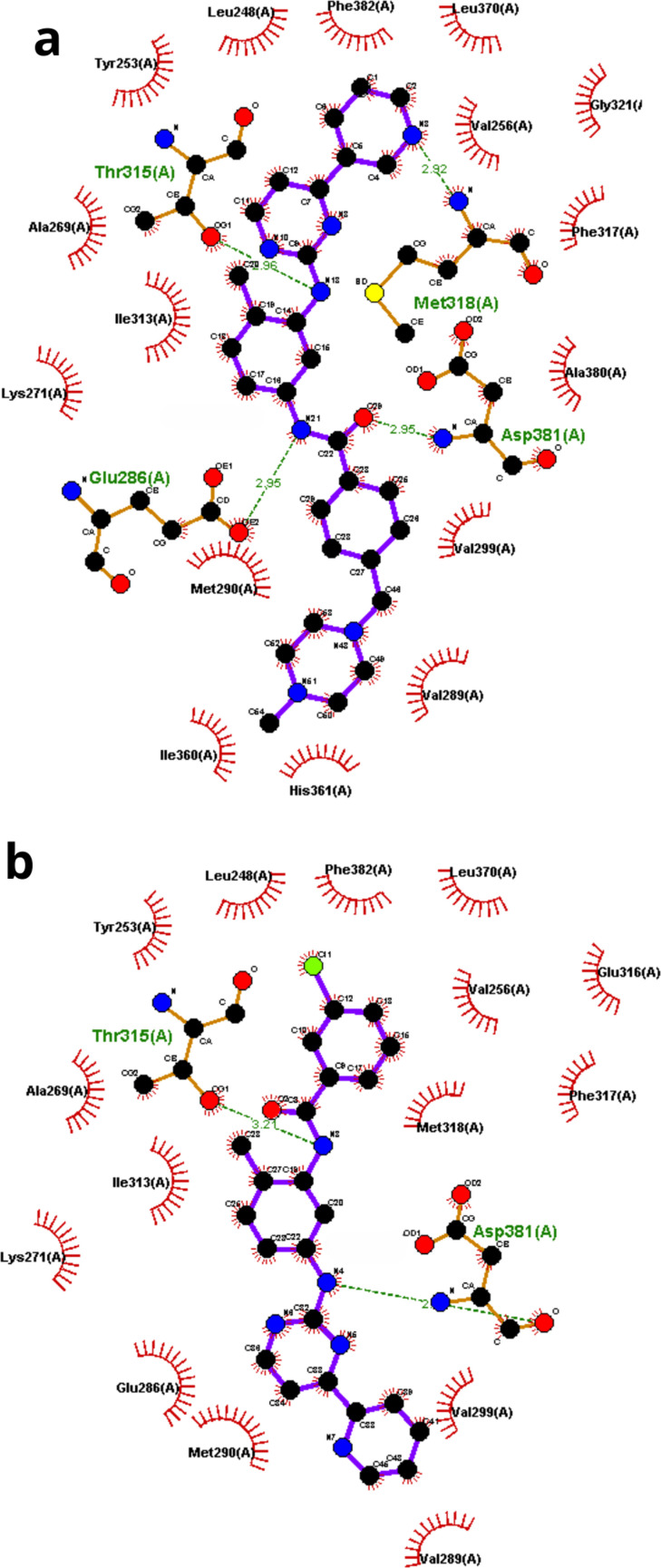
Diagram of the interaction with the active site of the subunit (A) of the ABL1 protein with (*a*) imatinib and (*b*) PAPP1. Atoms involved in hydrogen bonding interactions are shown in blue (nitrogen), red (oxygen), black (carbon) and yellow (sulfur).

Calculations were performed using AutoDock Vina software [32]. The configurations of the active site with imatinib and PAPP1 are illustrated in figure 8 using the graphical interface LigPlot+ [33].

The results suggest that PAPP1 can interact with the same protein pocket as imatinib, albeit in an opposite orientation. While PAPP1 forms fewer hydrogen bonds compared with imatinib, it shows significant interactions with key residues such as Thr315 and Asp381. Additionally, PAPP1 engages in hydrophobic interactions with surrounding residues despite having a smaller surface molecular area than imatinib. The ligand efficiency of PAPP1 (−0.38 kcal mol^−1^) surpasses that of imatinib (−0.35 kcal mol^−1^), with ligand efficiency defined as the ratio of docking score to molecular size [34,35].

The absence of a bulky group attached to the acyl ring in PAPP1 is a key factor influencing its interaction profile within the active pocket. This is reflected in the calculated free energy of interaction, which is Δ*G* = −11.8 kcal mol^−1^ for PAPP1, compared with −12.9 kcal mol^−1^ for imatinib. A notable feature of PAPP1 is the positioning of the chlorine atom in ring (E), located adjacent to an aromatic residue such as Phe382, suggesting a potential halogen–aromatic interaction similar to that observed in the crystal form. This interaction could potentially enhance the residence time of PAPP1 in the protein cavity [36].

Interestingly, the inverted conformation of PAPP scaffold compared with the experimental and redocked conformation of imatinib raises the potential of overcoming resistance issues mechanisms associated with known mutations. Furthermore, the nitrogen position in the pyrimidine ring (A) in PAPP1 constrains the *syn* conformation relative to ring B, a feature that enhances the selectivity of PAPP1.

These results indicate that PAPP1 is a promising candidate for further optimization and development as a TKI in future studies.

### 2.8. Drug type and ADME-Tox properties

Pharmacokinetic properties were evaluated using the Web-based SwissADME server, with the results summarized in table 3 [37]. The analysis shows that PAPP1 exhibits pharmacokinetic characteristics comparable to those of approved drugs such as imatinib and nilotinib, particularly in terms of drug-likeness and bioavailability.

**Table 3.** Pharmacokinetic computed properties.

molecule	MW	cLogP	cLogS	TPSA	HBA	HBD	RB
PAPP1	429.90	4.34	−5.95	79.80	4	2	7
imatinib	493.69	3.38	−5.05	86.28	6	2	8
nilotinib	529.52	4.63	−6.46	97.62	8	2	8

Key pharmacokinetic parameters, including hydrophobicity (as measured by cLogP and topological polar surface area, TPSA), the number of HBD and acceptors (HBA), the number of rotatable bonds (RB), and molecular weight (MW), indicate that PAPP1 is a promising candidate for further optimization aimed at improving its drug-like properties and oral bioavailability.

Further evaluation using ADMETlab 3.0 provided insights into the absorption, distribution, metabolism, excretion, and toxicity (ADME-Tox) features of PAPP1 [38]. Detailed data are available in electronic supplementary material, table S3. The compound demonstrated favourable quantitative estimates of drug-likeness (QED), surpassing those of reference compounds and showed comparable synthetic accessibility scores (SAscore). Regarding absorption, PAPP1 displayed superior Caco-2 permeability compared to imatinib and nilotinib, alongside the active potential for human intestinal absorption. However, its oral bioavailability fraction was below 50%, contrasting with the higher values reported for the reference drugs.

For distribution-related properties, PAPP1 was unlikely to inhibit P-glycoprotein (P-gp) and exhibited plasma protein binding probabilities similar to those of nilotinib. Its predicted ability to cross the blood–brain barrier was comparable to that of the reference compounds. In terms of metabolism, PAPP1 was predicted to function as an inhibitor and substrate of CYP1A2, while serving only as an inhibitor—but not substrate—of CYP2C19, CYP2C9, CYP3A4, and CYP2B6. It was neither an inhibitor nor a substrate of CYP2D6 or CYP2C8. Notably, the compound displayed human liver microsomal (HLM) stability values between those of imatinib and nilotinib, albeit with a relatively short half-life. Its clearance rates, however, were promising and comparable to the reference drugs.

Regarding toxicity, the performance of PAPP1 was similar to that of imatinib and nilotinib, with moderate risks identified for hERG blockade, human hepatotoxicity (H-HT), and drug-induced liver injury (DILI). Ames mutagenicity and rat oral acute toxicity also showed medium values, aligning with the reference compounds.

Finally, we have reported the synthesis and detailed characterization of PAPP1, demonstrating its viability in terms of atomic economy, predicted intermolecular interactions with the BCR::ABL1 oncoprotein, and its stability as evidenced by its UV–visible spectra and solid-state conformation. Future studies will focus on the biological evaluation of PAPP1 against the oncoprotein and its clinically relevant mutations, as well as exploring potential molecular optimizations to enhance its efficacy and selectivity.

## 3. Conclusions

This study successfully synthesized and characterized the novel PAPP1 compound, a PAPP derivative with potential as a TKI. Crystallographic analysis revealed a nearly coplanar arrangement of key rings, supported by hydrogen bonding and halogen intermolecular interactions that stabilize the crystal structure. HS analysis confirmed that hydrogen bonding, vdW forces and π–π interactions contribute to molecular layer stability.

Theoretical DFT calculations indicated a small HOMO–LUMO energy gap, suggesting high chemical reactivity, while MEP analysis identified strong nucleophilic and electrophilic regions crucial for intermolecular interactions. Energy frameworks show that dispersion and electrostatic interactions play a central role in stabilizing the crystal structure.

Molecular docking suggested potential interaction with the ABL1 TK domain, forming significant interactions with key residues such as Thr315 and Asp381. The higher ligand efficiency and favourable free energy of interaction compared to imatinib suggest binding potential.

Simulated pharmacokinetic and pharmacodynamic analysis confirmed that PAPP1 exhibits favourable drug-like and balanced ADME-Tox properties, aligning well with approved TKIs and indicating its promise for oral delivery.

PAPP1 is a promising candidate for further development as a TKI, with well-characterized crystallographic and theoretical properties supporting its potential therapeutic efficacy in targeting CML. Additionally, it improves atomic economy and maximizes intermolecular interactions, according to solid-state conformation studies. Future studies will involve biological testing against the BCR::ABL1 oncoprotein and its biologically relevant mutations, as well as the design, synthesis, and evaluation of analogues to optimize therapeutic potential.

## 4. Experimental section

### 4.1. Synthesis and crystallization

**Scheme 1.**
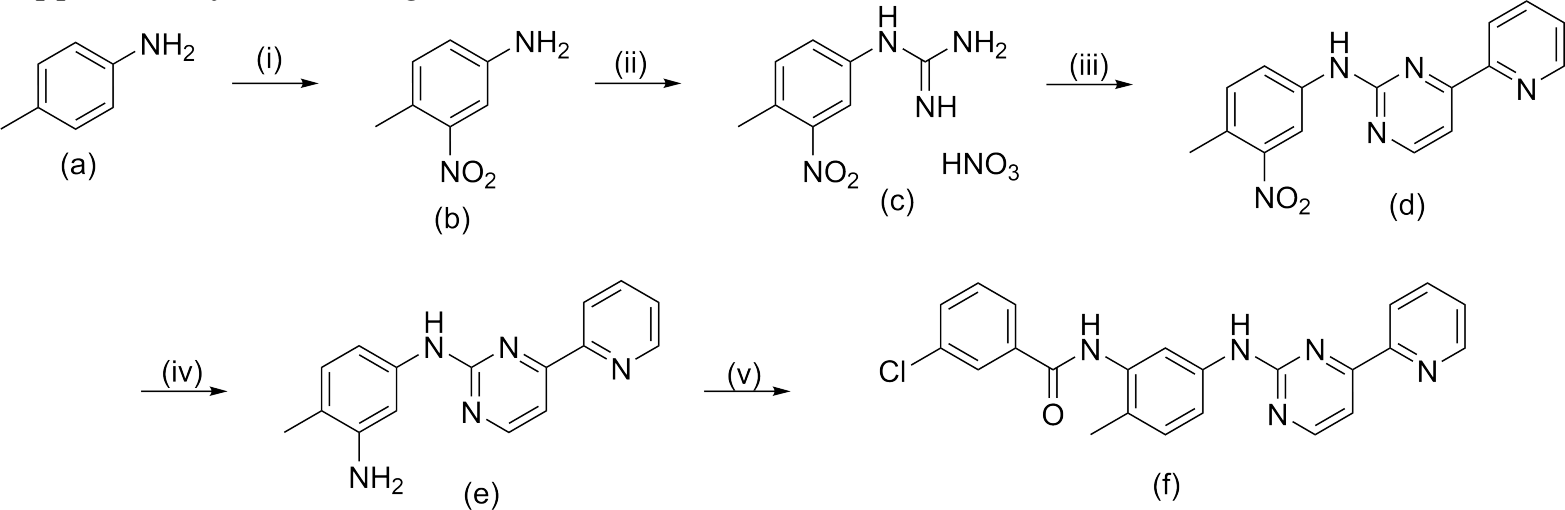
Synthetic route for the formation of amide derivatives of 2-phenylaminopyridylpyrimidine. (i) H_2_SO_4_, HNO_3_, 2 h, −10°C; (ii) HNO_3_, NC–NH_2_, PropOH, H_2_O, 30 h, 80°C; (iii) NaOH, 3-dimethylamino-1-(2-pyridyl)-2-propen-1-one, PropOH, 30 h, 75°C; (iv) SnCl_2_·H_2_O, HCl, 48 h, 0°C; (v) 3-chlorobenzoyl chloride, CHCl_3_, 3 h, room temperature.

In the initial step, the synthesis of amides derived from 2-phenylaminopyridylpyrimidine (template (d)) is proposed, utilizing a well-established and optimized synthetic route (Scheme 1). This route involves the formation of pyrimidines through a previously described cyclization process [17,39,40]. The first step (i) involves the nitration of *p*-toluidine (a) using nitric acid in sulfuric acid. This reaction proceeds via electrophilic substitution and is highly selective due to the orientation effects of the deactivated protonated amine (*meta*-orientation) and the methyl group (*ortho*-orientation) [41]. Upon neutralization of the acid, the formation of a precipitate with the characteristic orange colour of nitrated compounds indicates the formation of product (b). This product was subsequently isolated and characterized using NMR and electron impact mass spectrometry (EI-MS) analysis (electronic supplementary material, figures S1–S4).

The nucleophilic addition of cyanamide to the nitrated compound (b) in a polar medium (*n*-propanol/water) and in the presence of an acid, which enhances the electrophilicity of cyanamide, resulted in the formation of phenyl guanidinium salt (c) [42]. Despite a decrease in the nucleophilicity of the amino group due to protonation [28], this salt was obtained in high yield and characterized by NMR, which displayed the expected characteristic signals (electronic supplementary material, figures S5−S7), and by EI-MS at 40 eV (electronic supplementary material, figure S8).

Product (c) was then reacted with the enaminone, 3-dimethylamino-1-(2-pyridyl)-2-propen-1-one, using *n*-propanol as a solvent, leading to the formation of the pyrimidine ring substituted at positions 2 and 4. This reaction is a modification of the dihydropyrimidine formation reaction developed by Traube and Schwarz, which involves the condensation of an α,β-unsaturated ketone, removal of dimethylamine, and dehydration to achieve aromaticity [43].

Template (d) was then reacted in an acidic medium with SnCl_₂_·H_₂_O as a reducing agent to produce amine (e) [44]. Both products, (d) and (e), were characterized using spectroscopic methods (electronic supplementary material, figures S9–S15). From amine (e), the target compound PAPP1 was synthesized through the acylation of the amino group with 3-chlorobenzoyl chloride [45]. PAPP1 was purified by recrystallization, characterized by NMR and EI-MS and its identity was confirmed (electronic supplementary material, figure S16–S19).

### 4.2. General procedure for the preparation of phenylaminobenzamides

100 mg of precursor (e) (0.36 mmol) ws dissolved in 1 ml of CHCl_3_ and dropwise mixed with 1 ml of a solution containing an equivalent of the corresponding substituted acid chloride in CHCl_3_. The mixture was stirred for 3 h at room temperature. A yellow solid was observed to form, which was filtered and washed with cold chloroform.

### 4.3. *N*-[2-Methyl-5-(4-pyridin-2-ylpyrimidin-2-ylamino)phenyl]-3-chlorobenzamide (PAPP1)

Strong yellow solid. Recrystallized in MeOH/1-PropOH 9:1. 87 mg (58%) was isolated. M.p. 205.3–207.2°C. ^1^H NMR (DMSO-*d*_6_, 400 MHz, δ ppm): 10.07 (s), 9.83 (s), 8.75 (d, *J* = 4.7 Hz, **Ar**), 8.64 (d, *J* = 5.1 Hz, **Ar**), 8.50 (d, *J* = 7.9 Hz, **Ar**), 8.09−7.95 (m, 4H, **Ar**), 7.73 (d, *J* = 5.1 Hz, **Ar**), 7.68 (dd, *J* = 7.9, 2.0 Hz, **Ar**), 7.59 (ddd, *J* = 8.0, 5.2, 2.8 Hz, 3H, **Ar**), 7.23 (d, *J* = 8.3 Hz, **Ar**), 2.21 (s, 3H). ^13^C{^1^H} NMR (DMSO-*d*_6_, 100 MHz, δ ppm): 163.95 (1C, **Ar**), 162.51 (1C, **Ar**), 159.77 (1C, **Ar**), 159.27 (1C, **Ar**), 153.14 (1C, **Ar**), 149.20 (1C, **Ar**), 138.31 (1C, **Ar**), 138.02 (1C, **Ar**), 136.57 (1C, **Ar**), 136.00 (1C, **Ar**), 133.30 (1C, **Ar**), 131.39 (1C, **Ar**), 130.51 (1C, **Ar**), 130.19 (1C, **Ar**), 127.43 (1C, **Ar**), 126.96 (1C, **Ar**), 126.41 (1C, **Ar**), 125.99 (1C, **Ar**), 121.63 (1C, **Ar**), 117.59 (1C, **Ar**), 117.32 (1C, **Ar**), 107.90 (1C, **Ar**), 17.30 (1C). MS (IE, 40 eV), *m*/*z* = 415.20 (41.66%), 416.20 (14.37%), 417.20 (15.29%) [M^+^].

### 4.4. X-ray crystallographic analysis

#### 4.4.1. Data collection and refinement details

Diffraction data for the PAPP1 compound wre collected using a Bruker AXS Enraf–Nonius KappaCCD diffractometer with Cu *K*α radiation (λ = 1.5418 Å). The data were corrected and solved using direct methods with SHELXS-97 [46] and refined by full-matrix least-squares methods on F² with SHELXL-2014 [47]. All hydrogen atoms, except H–N and Hw–O, were placed in geometrically idealized positions: C–H = 0.95 Å and C–H (methyl) = 0.98 Å. These hydrogen atoms were refined using a riding model approximation with U_iso(H) = 1.2 U_eq (for ring atoms) and U_iso(H) = 1.5 U_eq (for methyl atoms). The H–N and Hw–O atoms were located from Fourier difference maps, and their coordinates were refined freely. The accuracy of the model was confirmed by low residuals in the final difference map, with peak and hole values of 0.290 e·Å⁻³ and −0.314 e·Å⁻³, respectively. Mercury software generated molecular and supramolecular graphics [48].

### 4.5. Computational details

The geometry optimization of PAPP1 was conducted using the B3LYP method, a three-parameter Becke hybrid DFT combined with Lee−Yang–Parr nonlocal correlation, in the gas phase [49–51]. The corresponding harmonic frequencies were calculated at the same level of theory to confirm that the stationary points are minima (no imaginary frequencies) using the GAUSSIAN09 program package [52]. All calculations, except for the energy-framework calculations on molecular pairs in the solid state, were performed at the 6-31G(d,p) level of theory. DFT with the B3LYP-D3/6-31G level of theory was employed for the energy framework calculations.

## Data Availability

Full crystallographic data have been deposited with the Crystallographic Data Centre (CCDC), reference number 2286964, which contains the supplementary crystallographic data for this paper. These data can be obtained free of charge via http://www.ccdc.cam.ac.uk/conts/retrieving.html (or from the CCDC, 12 Union Road, Cambridge CB2 1EZ, UK; Fax: +44 1223 336033; E-mail: deposit@ccdc.cam.ac.uk). Additionally, experimental crystallographic data, along with spectroscopic information, are available in the Dryad Data Repository [53]. Supplementary material is available online [54].
